# Stable speech BCI performance during slow progression of ALS: A longitudinal ECoG study

**DOI:** 10.21203/rs.3.rs-9156039/v1

**Published:** 2026-03-25

**Authors:** Ziwei Ouyang, Kalan Walmsley, Shiyu Luo, Donna Tippett, Kimberley Wyse-Sookoo, Matthew Fifer, Mariska J. Vansteensel, Miguel Angrick, Nick Ramsey, Nathan E. Crone

**Affiliations:** Johns Hopkins University; École Polytechnique Fédérale de Lausanne; Johns Hopkins University; Johns Hopkins University School of Medicine; Johns Hopkins University; Johns Hopkins University Applied Physics Laboratory; University Medical Center Utrecht; Johns Hopkins University School of Medicine; University Medical Center Utrecht; Johns Hopkins University School of Medicine

## Abstract

**Background:**

Electrocorticographic (ECoG) speech brain-computer interfaces (BCIs) show promise for restoring communication in amyotrophic lateral sclerosis (ALS), but the long-term stability of speech-related neural signals and decoding performance during disease progression remains unclear. We tracked signal characteristics and decoding over 25 months in a participant with ALS to determine how high-gamma (HG, 70–170 Hz) activity changes over time and whether these changes affect offline speech decoding.

**Methods:**

We implanted two 8×8 subdural ECoG grids over left sensorimotor cortex (SMC) in a participant with slowly progressive bulbar variant ALS. Across 25 months, the participant performed an overt syllable-repetition task (12 consonant-vowel tokens) during simultaneous ECoG and audio recording. We quantified HG activation ratio (ActR), spectral signal-to-noise ratio (SNR; HG/HF, where HF = 300–499 Hz), and peak z-scored HG responses. Speech acoustics were evaluated using first/second formants (F1/F2) and the triangular vowel space area (tVSA). Offline EEGNet-based decoders were assessed in two stages: models trained on post-implant months 1–6 were tested on months 7–25, while models trained on stabilized data (months 7–11) were tested on the remaining period (months 12–25). Electrode-level saliency assessed spatial contributions to decoding.

**Results:**

Acoustic analyses showed a significant reduction in tVSA over two years (−44.6 Hz^2^/day; *P* < 10^−7^), consistent with mild intelligibility decline. Neural metrics (ActR and SNR) followed a biphasic trajectory: increasing during the first 6 months, after which ActR stabilized (0.041%/day; *P* = 0.13), and SNR declined gradually (−0.46%/day, *P* < 10^− 4^). The model trained on months 1–6 achieved 55.7% accuracy (chance: 8.33%), but performance declined over time (−0.019%/day; *P* = 2.1×10^−4^). Conversely, the model trained on months 7–11 achieved higher accuracy (65.9%) on subsequent data with no significant temporal decline (*P* = 0.23).

**Conclusions:**

Speech-related HG features exhibited an initial unstable period followed by a long-term gradual SNR reduction, potentially reflecting disease progression. Models trained after signal stabilization generalized robustly to data recorded over a year later. These findings confirm that despite reduced absolute HG power and mild acoustic degradation of speech, cortical features remain stable enough to support durable ECoG speech BCIs without frequent recalibration. These findings will motivate future adaptive calibration algorithms that account for slow signal changes while leveraging stable spatial representations in ventral SMC.

**ClinicalTrials.gov Identifier:**

NCT03567213

## Background

For individuals with amyotrophic lateral sclerosis (ALS), the loss of communication severely compromises quality of life and social participation.[1–3] To retain the naturalness and speed of speech, recent efforts have shifted toward directly decoding attempted speech from cortical activity.[4–6] State-of-the-art speech neuroprostheses can now generate text or synthesized audio with high accuracy and minimal calibration, approaching the speed of natural speech.[7, 8] This direct approach offers a clinically promising path to restore rapid, intuitive communication for patients with severe paralysis. [3, 9]

Electrocorticography (ECoG) has been used to effectively decode brain-to-speech from articulatory representations in ventral sensorimotor cortex (vSMC).[5, 10–14] Compared to microelectrode arrays, ECoG has the potential for less cortical tissue disruption and more stable long-term recordings.[15] To decode intended behavior, ECoG BCIs typically utilize broadband high-gamma activity (~ 60–200 Hz). Broadband high-gamma is tightly correlated with the aggregate firing rates of neural populations within the effective recording volume of cortical surface electrodes.[16, 17] By measuring aggregate firing rates, ECoG signals are theoretically less susceptible to the dropout of individual neurons over time.

Previous work has shown that ECoG recordings from primary motor cortex can support stable and reliable multi-year at-home BCI use for communication by selecting icons on a computer screen in an individual with locked-in syndrome due to ALS.[18, 19] Stability was also observed in controlled exoskeleton tasks that utilize upper limb ECoG-based BCIs.[20, 21]

While these results are promising, it is unclear whether the long-term stabilization of signaling and decoding observed in upper-limb tasks applies to speech regions of SMC. Second, unlike paralysis resulting from brainstem stroke or other nonprogressive etiologies, the progressive nature of ALS may pose unique challenges to the long-term stability of BCI systems. As brain atrophy progresses and motoneurons are lost, neural dynamics during speech production may change, potentially affecting decoding accuracy trained on neural dynamics at a different stage of the disease.[19, 22] Existing studies have been primarily limited to short-term (weeks to months) or at most one-year observation windows,[11, 23] whereas the time course of ALS can span several years.[24, 25] This mismatch in time scales limits our ability to understand the long-term effects of disease progression on speech-related neural activity. Prior work showed that HG responses in the SMC of ALS patients remain relatively stable over a 6-month period.[23] Furthermore, it has also been reported that patients with ALS may be influenced by a gradual decline in HG responses over larger time scales.[18, 19] These complex changes may require longer observation windows to capture, highlighting the critical value of longitudinal studies over two years.

In this study, we measured and quantified changes in ECoG HG signals recorded from vSMC in a patient living with ALS over a period of 25 months. At the same time, we investigated how the performance of BCI speech decoding models changed over time. We used acoustic speech recordings to observe changes in articulation through metrics based on formant frequencies, as well as perceptual assessments from a speech pathologist, to determine whether changes in neural activity are reflected in different speaking behaviors. Our longitudinal analysis provides further insights into the stability of ECoG signals, how they change in relationship to the progression of ALS symptoms, and to what extent those changes can affect BCI decoding models trained on earlier representations.

## Methods

### Standard Protocol Approvals, Registrations, and Patient Consents

**Figure 1:**
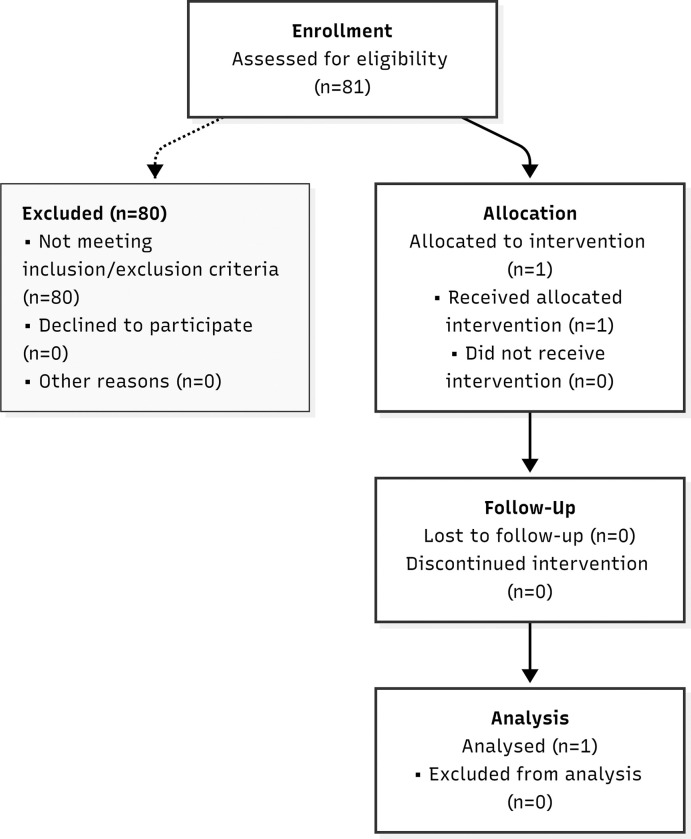
CONSORT flow diagram of participant screening and enrollment. The diagram illustrates the flow of participants through the study phases. As of October 1, 2024, a total of 81 candidates were assessed for eligibility. Eighty candidates were excluded for failing to meet the strict inclusion and exclusion criteria. Ultimately, 1 participant was enrolled, received the allocated intervention, and was included in the final data analysis.

The clinical trial was conducted under an investigational device exemption from the U.S. Food and Drug Administration (FDA). The protocol was approved by the Johns Hopkins School of Medicine Institutional Review Board (JHM IRB, protocol number: IRB00167247). Additionally, the study was registered on ClinicalTrials.gov under the identifier NCT03567213 (CortiCom Clinical Trial). As of October 1, 2024, 81 candidates were screened, resulting in one enrolled participant (Fig. 1). Eligible participants included individuals with impaired speech and upper-extremity motor function (additional information about the participant is available in Supplementary Appendix 1). Written informed consent was obtained from the participant. The study adhered to the ethical guidelines outlined in the Declaration of Helsinki.

### Implanted device and technical details

**Figure 2:**
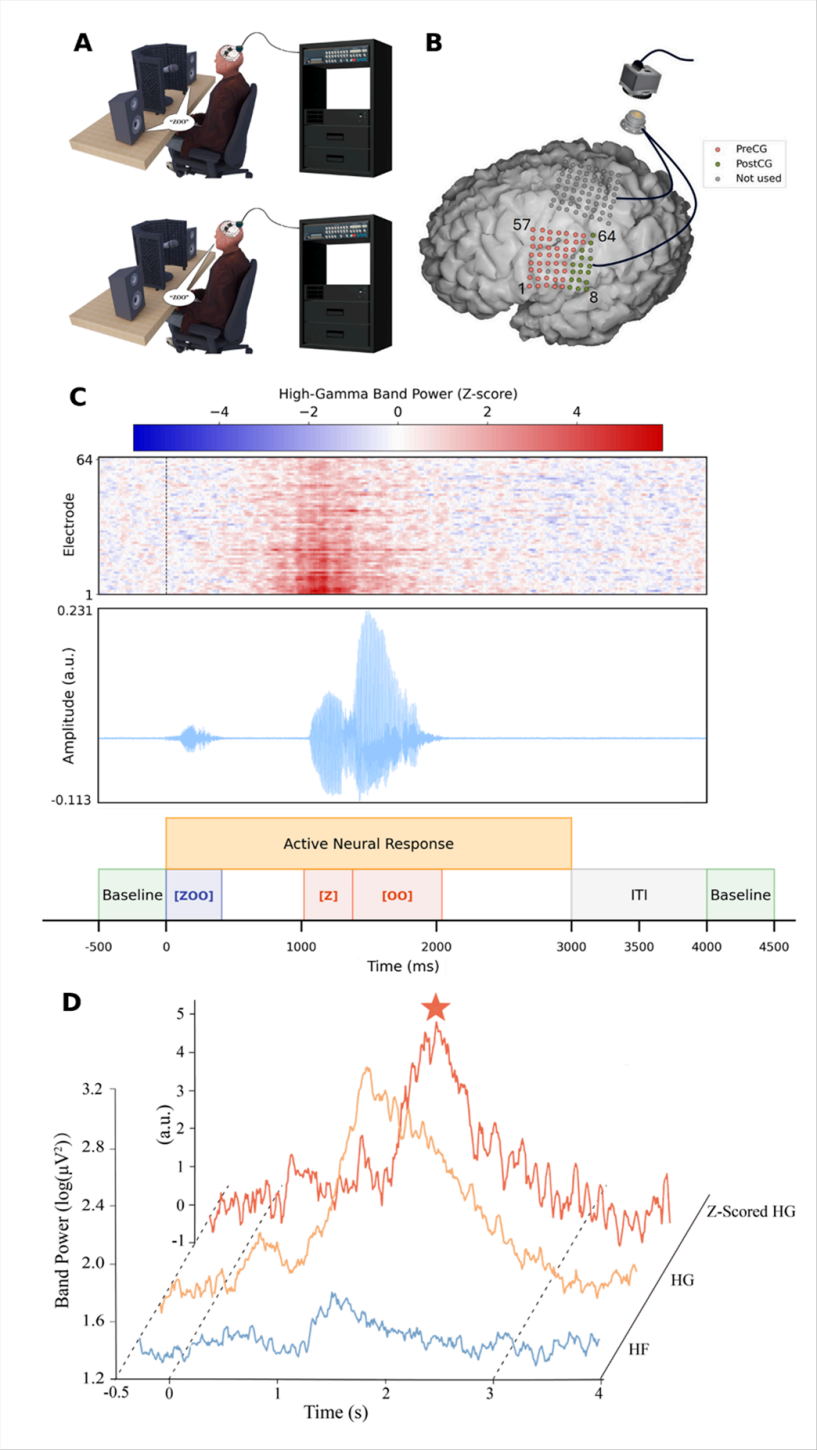
Experimental setup, electrode placement, and neural response dynamics during a syllable repetition task. (A) During the task, the patient was instructed to overtly repeat target syllables (e.g., “BAH”) in response to spoken cues, while ECoG and audio signals were recorded synchronously. (B) ECoG grid placement on the SMC of the left hemisphere, covering pre-central (red) and post-central (green) gyri. Electrodes not used for analysis are shown in gray. (C) Top: Z-scored HG activity from all analyzed electrodes, normalized against the pre-stimulus baseline period (before the dashed line), with red indicating elevated activity. Middle: Normalized acoustic waveform of the syllable “ZOO”. Bottom: Task timeline showing baseline, stimulus epochs (“ZOO”), consonant-vowel segments (“Z”, “OO”), intertrial interval (ITI, can be any length between 0–1s), and the window of active neural response. (D) Representative electrophysiological activity from channel 8, where the high-frequency (HF) and high-gamma (HG) power are the grand average across all syllables and trials, while the Z-scored HG power shows an example single-trial response to the syllable “ZOO,” with its peak marked by a star and baseline (−0.5 to 0 s) and activation (0 to 3 s) periods demarcated by dashed lines.

The Cortical Communication (CortiCom) System (Fig. 2A, B) used for the study consisted of two 8 × 8 subdural cortical ECoG grids (PMT Corporation, USA) with a 128-channel percutaneous Neuroport connector (Blackrock Neurotech, USA). Each ECoG grid contained 64 platinum-iridium electrodes (2 mm diameter, 0.76 mm thickness) embedded in a flexible silicone substrate (total area 12.11 cm^2^, 36.6 mm × 33.1 mm) at 4 mm center-to-center spacing (Fig. 2B). Two subdural reference wires were implanted superficially to the grids (Fig. 2B).

The investigational device was implanted with an awake craniotomy under local anesthesia and light sedation, in part to avoid neuromuscular blockade. Both ECoG grids were implanted over SMC in the left hemisphere, covering speech and upper limb areas. Postoperative CT-MRI co-registration confirmed anatomical positioning of the device.

### Task and Experiment Design

We used a syllable repetition task to elicit speech-related neural responses. For each experiment block, the participant was instructed to repeat consonant-vowel syllables generated by text-to-speech and audibly presented via loudspeakers. We chose 12 syllables (e.g., “BAH,” “ZOO,” etc.) with diverse phonetic features,[26] each pseudo-randomized to be repeated 5 times/block with a random stimulus interval of 3.5 to 4.5 seconds (total block duration of approximately 5 minutes, including a 30-second rest period before task onset; Fig. 2C). We performed up to 3 independent sessions per week (166 sessions in total), continuously accumulating the paired speech-ECoG dataset. Since this study focuses on speech as a control signal, we excluded all electrodes covering the upper limb region from subsequent analyses.

### Data Preprocessing

ECoG signals were acquired using the NeuroPort system (Blackrock Neurotech) and downsampled to 1 kHz for analysis. Synchronized high-fidelity audio was recorded to align speech tasks with neural data. Detailed acquisition hardware and audio synchronization protocols are provided in Supplementary Methods 1.

We performed impedance testing weekly with the impedance detector in the Blackrock Central software suite. Since the system only provides specific values for electrodes with impedances higher than 15 kOhm, tracking changes over time in detail for low-impedance electrodes was not possible. We excluded electrodes with persistently high impedance (> 15 kOhm, electrodes 19, 38, and 48) and with abnormal signals on visual inspection (electrode 52) from this study. The final electrodes included in the analysis were 60 electrodes from the lower grid covering vSMC. Data from 6 sessions were excluded from the analysis due to anomalies. We also used tuning fork experiments to demonstrate that the recorded ECoG signals were not contaminated by acoustic artifacts (Supplementary Methods 2).

Based on the data from the average spectrograms of all available sessions (Supplementary Fig. 1), we determined the boundary between the high gamma band (HG, 70–170 Hz) and the high-frequency (HF) noise floor (HF, 300–499 Hz). The HF band was further identified as noise using the established criteria from previous research.[23, 27] We extracted the HG and HF signals using 8th-order Butterworth IIR bandpass filters. After extracting the HG band, we employed a cascaded IIR filter at 118–122 Hz to remove the first harmonic of the line noise at 120 Hz. Finally, we calculated the log power of these signals within 50ms bins.

### Evaluation metrics

We first calculated the trial-averaged HG power and HF power (in dB) for all electrodes during resting (baseline) and active periods in each session (Fig. 2D). The baseline period was defined as 0.5 seconds before stimulus onset for each trial (Fig. 2D). The active period was defined as 3 seconds after stimulus onset, including auditory stimulus processing, speech planning and active vocalization.[28] By visually checking time-aligned acoustic waveforms, we verified that the participant did not need more than 3 seconds to repeat a syllable, and we also confirmed the delineation of baseline and active periods using trial-averaged HG responses across all electrodes, which lasted no more than 3 seconds (Supplementary Fig. 2).

We followed the definitions of activation ratio (ActR) and spectral signal-to-noise ratio (SNR) from previous work ^22^,

#(1) $${SNR}_{\text{baseline}\;\text{or}\;\text{active}}=10\log_{10}\frac{{PowerHC}_{\text{basaline}\;\text{or}\;\text{acsure}}}{{Power}_{{HP}_{\text{baseline}\;\text{or}\;\text{active}}}}$$

#(2) $$ActR=10{log}_{10}\frac{{\text{Power}}_{{HC}_{\text{actlre}}}}{{\text{PowerHG}}_{\text{bastline}}}\text{.}$$

Power_HG_ and Power_HF_ are the corresponding band-power trial averages. We modified the definition of the peak HG response compared with earlier work to avoid the effects of changes in peak latency.[23] We normalized the HG power for each trial by a Z-score and extracted the peaks from the normalized HG responses within the active period as the HG response peaks, and then averaged all the peaks in each session (Fig. 2D),

#(3) $$\begin{array}{c} {HG}_{\text{peak}}=max\left(\frac{{log}_{10}\left({Power}_{{HG}_{\text{active}}}\right)-\bar{b}}{std(b)}\right),\\ b=\mathrm{baseline}\;HG\;\mathrm{for}\;\mathrm{each}\;\mathrm{trial}. \end{array}$$

HG peak response Z-score indicates how many standard deviations above the baseline activity this peak is relative to the baseline signal.

### Acoustic analysis

The evolution of the patient’s speech quality throughout the two-year study was investigated to complement the neural signal analysis. The triangular vowel space area (tVSA) was the primary metric used to evaluate speech quality, as it correlates with speech intelligibility.[29] Specifically, this area refers to the triangle formed by the locations of the first and second formants (F1, F2) for the /a/, /u/, and /i/ vowels. Reductions in VSA have been shown to correlate with reductions in intelligibility.[29–31] Vowels were manually segmented from the previously mentioned syllable repetition task. The corresponding F1 and F2 were then extracted using Praat.[32] The F1 and F2 frequencies for a given vowel segment were taken to be the medians of the formant samples.[33] The (F1, F2) locations used to compute the tVSA were estimated for each measurement day by calculating the median across F1 and F2 for each vowel class (/a/, /u/, /i/). Subsequently, tVSA was computed using,

#(4) $$A=|\frac{1}{2}\underset{T_{1}}{\underset{︸}{\left[F_{1}^{\text{a}}-F_{1}^{\text{u}}\right]}}\cdot \underset{T_{2}}{\underset{︸}{\left[F_{2}^{\text{i}}-F_{2}^{\text{a}}\right]}}-\frac{1}{2}\underset{T_{3.}}{\underset{︸}{\left[F_{1}^{\text{a}}-F_{1}^{\text{i}}\right]}}\cdot \underset{T_{4.}}{\underset{︸}{\left[F_{2}^{\text{u}}-F_{2}^{\text{a}}\right]}}|.$$

We then assessed the statistical significance of the changes in tVSA (see detailed derivation in Supplementary Methods 3).

### Syllable classification model

We developed a deep convolutional neural network (CNN) based on the EEGNet architecture to decode twelve syllables from ECoG signals.[34] To improve the feature extraction and decoding performance, we integrated residual blocks with Squeeze-and-Excitation (SE) attention mechanisms into the network architecture.[35, 36] This design allowed the network to amplify feature channels with richer information adaptively[35] and to optimize gradient flow in deep networks.[36]

Input data comprised HG (70–170 Hz) power from 60 cortical channels, extracted from a 3-second post-cue window. Signals were baseline-normalized (−0.5s to 0s) to reduce signal drift and inter-trial variability. We divided the data into training (40 days), validation (the subsequent 10 days), and independent test sets (the remaining days) based on recording dates to assess decoding stability. Detailed model architecture diagrams, specific parameter settings, and training protocol are included in Supplementary Methods 4.

### Statistical Analysis

All data in this article are presented as raw values or box plots. Signal trends were analyzed using linear regression based on least squares approximation. Here, we used Python 3.10 and scikit-learn 1.7.1. A p-value less than 0.05 was considered statistically significant. We analyzed temporal trends in neural signals and decoding performance using linear regression, and assessed their significance with a two-tailed t-test and the Holm-Bonferroni correction. Group comparisons were performed using Welch’s t-test for decoding accuracies and a non-parametric Wilcoxon signed-rank test for electrode slope distributions. For electrode-wise analyses involving multiple comparisons, we applied the Benjamini-Hochberg procedure to control the False Discovery Rate (BH-FDR) at q = 0.01.

## Results

### HG responses were stable after 6 months of implantation

**Figure 3:**
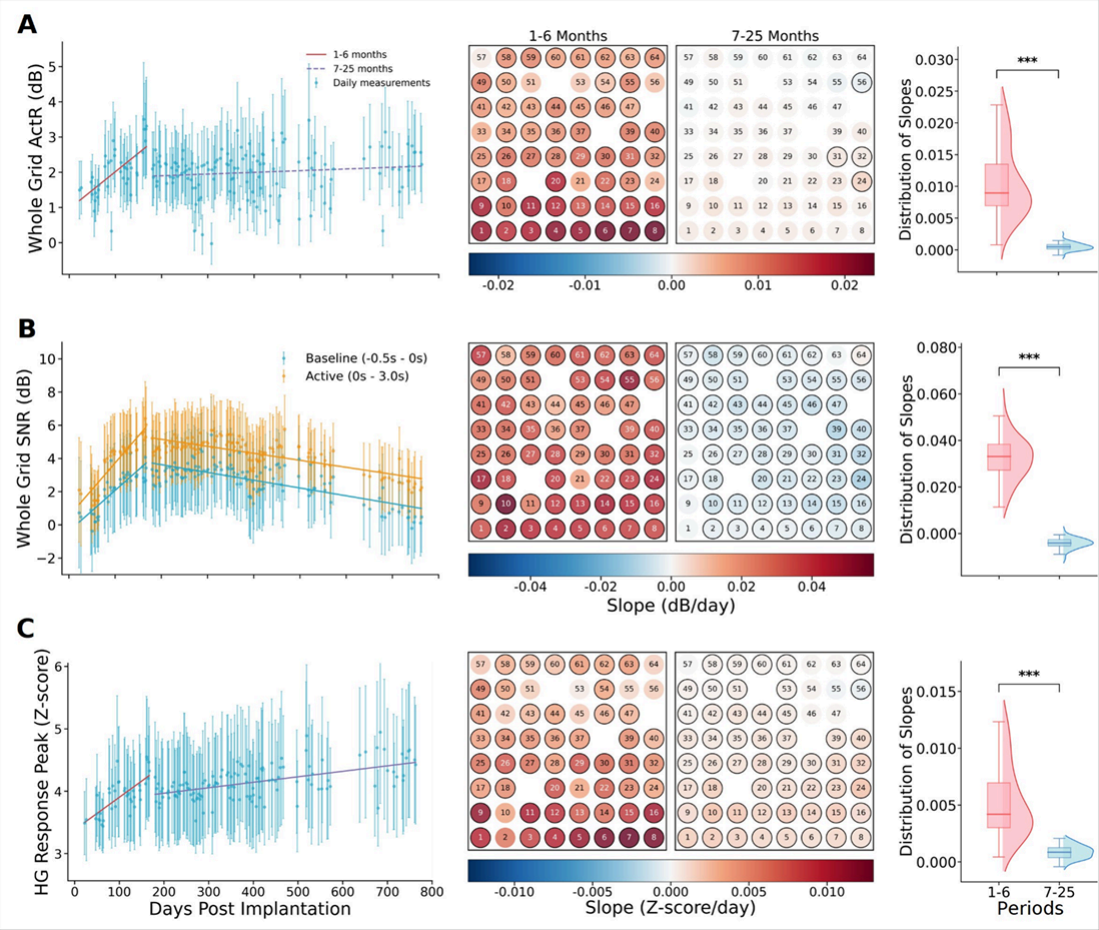
Longitudinal changes in high gamma signal metrics across electrodes over 25 months post-implantation. One key metric for each row. (A) ActR (dB) showed early increases followed by stable activation. (B) SNR (dB) initially improved and declined gradually. (C) HG response peak (Z-score) exhibited increasing trends in early and late phases. The left panels display time-series data (cyan or orange dots, represent mean ± SD across all 60 analyzed electrodes) with trend lines fitted over the early (1–6 months, red) and late (7–25 months, purple) phases. Solid lines indicate significant trends (α = 0.05, two-tailed t-test), while the dashed line indicates non-significance. The middle panels show spatial distributions of slope values across the 8×8 electrode grid. Electrodes with significant trends are outlined in black (*q* = 0.01, two-tailed t-test, with BH-FDR control, k = 60). The right panels display the distribution of slope values across the two periods, with group differences assessed using the Wilcoxon signed-rank test (*** *P* < 0.001).

We investigated the temporal evolution of ActR during 763 days following implantation of the CortiCom device. Periodic measurements of ActR across the entire grid exhibited a biphasic pattern (Fig. 3A, left). During the initial 1–6 months, ActR displayed an increasing trend (slope = 0.88%, *P* = 1.09×10^− 5^), rising from approximately 1.2 dB to 2.6 dB (Fig. 3A, left). In contrast, the 7–25-month period showed greater stability with less drift (slope = 0.041%, *P* = 0.13), maintaining ActR values around 2.0 dB despite session-level fluctuations (Fig. 3A, left). An electrode-wise analysis revealed spatial heterogeneity across the ECoG grid, with a more pronounced rising trend in ActR in the lower two rows of electrodes where speech-related HG responses were greatest (Fig. 3A, middle). 88.3% of the channels showed an increased ActR in the early phase (*P* < 0.0080 after BH-FDR correction). Notably, 93.3% of the channels exhibited no significant change in the later stages (*P* ≥ 0.00056 after BH-FDR correction). The distribution of electrode slopes narrowed over time (median slopes: early = 0.75%; late = 0.02%), with early slopes being higher than late slopes for every electrode (*P* = 1.63 × 10^− 11^, Fig. 3A, right). These findings indicate that HG activations can take several months to adjust after implantation, which may be related to changes in the electrode-tissue interface.[37]

### High gamma SNR gradually declined after 6 months of implantation

We defined high gamma SNR as the ratio of HG power to the noise floor estimated from HF power. We calculated SNR separately for active and baseline periods. SNR during the speech (active) period was consistently higher than that of the baseline period. Both showed biphasic longitudinal trends (an increasing trend followed by a decreasing trend) with similar magnitudes of change (Fig. 3B, left; Supplementary Table 1). During the first 6 months, SNR during the active period rose from approximately 1 dB to 6 dB, then gradually decreased to approximately 3 dB by day 763 (Fig. 3B, left). Electrode-wise analysis revealed a more uniform pattern of SNR evolution across the electrode grid compared to the ActR, with no evident spatial heterogeneity (Fig. 3B, middle). The analysis of the slope distribution shows that all slopes within the first six months were positive (median = 0.032), whereas those in the late period were predominantly negative (median = −0.004; Fig. 3B, right). These changes in SNR likely resulted from changes in HG band power in both baseline and active periods, as HF noise remained constant across all recording sessions (Supplementary Fig. 3, Supplementary Table 1).

### HG response peaks gradually increased over time

Next, we quantified longitudinal trends in the peak speech-related HG responses, measured as Z-scores relative to the resting baseline. This peak represents the moment of highest neural activity relative to rest. This typically occurred during the vowel segment of the participant’s speech.[26] Analysis of daily measurements revealed a gradual increase in the HG response peak over time (Fig. 3C, left). The HG peak significantly increased during the first six months, from approximately 3.3 to 4.0 standard deviations. This upward trajectory continued later, though with a reduced slope (slope = 0.084%, *P* = 8.11 × 10^− 12^), with Z-scores reaching around 4.2 by day 763. Examination of the individual electrodes showed a consistent increasing trend across nearly the entire electrode grid; however, this trend was not statistically significant for the dorsal posterior electrodes over the 7–25-month period (Fig. 3C, middle). This electrode-wise consistency was also reflected in the distribution of trend slopes, which remained predominantly positive across both periods and were higher during the early stage (1–6 months: median = 0.5%; 7–25 months: median = 0.1%, *P* = 1.63 × 10^− 11^; Fig. 3C, right).

### Disease progression had mild effects on speech intelligibility

**Figure 4:**
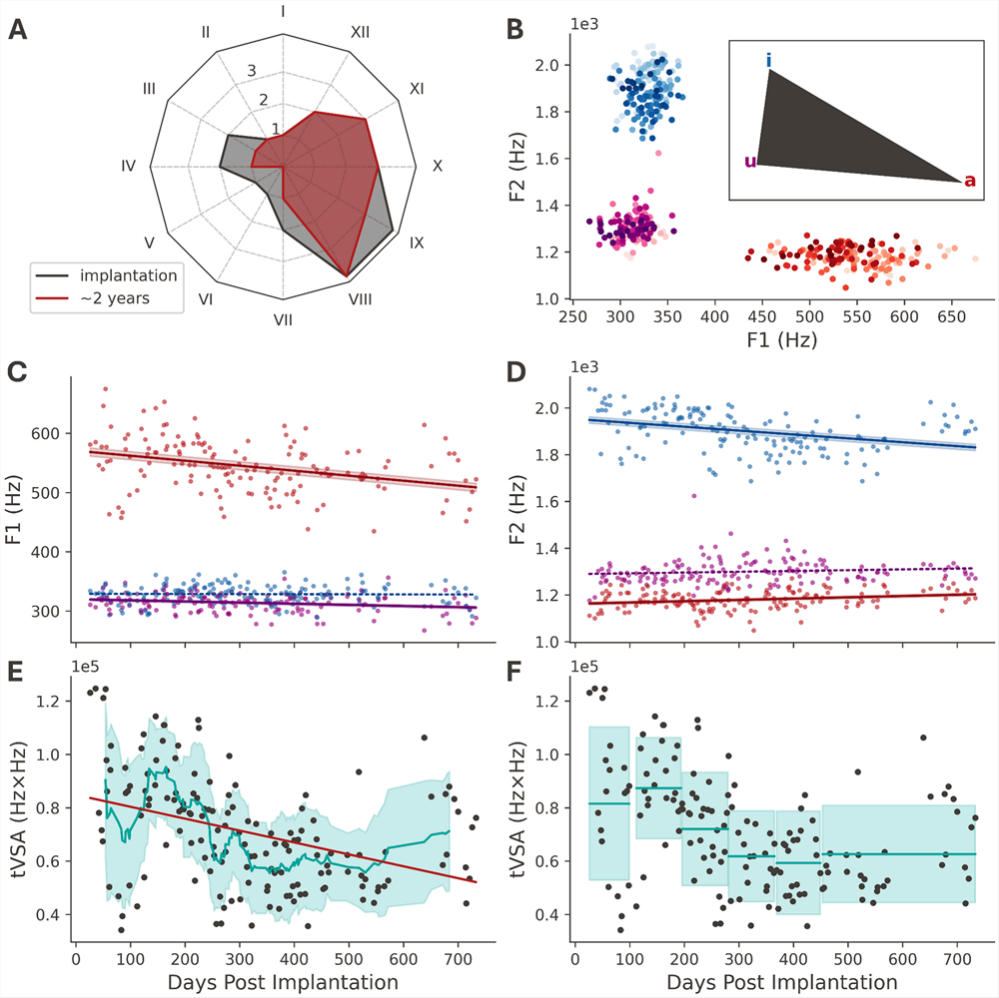
Two-year evolution of tVSA. (A) Radar plot of patient assessment on the ALSFRS-R scale at implantation and about two years later. I. Speech, II. Respiratory insufficiency, III. Swallowing, IV. Handwriting, V. Cutting food and handling utensils, VI. Dressing and hygiene, VII. Turning in bed & adjusting bedclothes, VIII. Walking, IX. Climbing stairs, X. Dyspnea, XI. Orthopnea and XII. Salivation. (B) Daily median vowel F1/F2 locations. The colors correspond to those of the vowels in the inset schematic. Darker shades in each group indicate an increase in the number of days after implantation. (C), (D) Median vowel F1 and F2 over time. Solid regression lines indicate significance with *α* < 0.0083, t-test, and Holm-Bonferroni correction. (C) The significant F1 slopes are −0.084 Hz/day for vowel /a/ and −0.019 Hz/day for /u/. (D) The significant F2 slopes are 0.055 Hz/day for /a/ and −0.166 Hz/day for /i/. d, e, tVSA over time. (E) The cyan line and confidence area represent the 13-day rolling mean and standard deviation of tVSA. The slope of the red regression line is −44.594 Hz^2^/day, *p* < 10^−7^. (F) The cyan lines and confidence areas reflect approximately 3-month periods, except for the last period, which encompasses around 6 months.

Over the 25-month study period, we observed slight changes in how our participant produced certain syllables. While ALS disease progression had noticeable effects on his ability to perform activities of daily living, as tracked by the ALSFSR-R scale in Fig. 4A, his speech was ranked “1” (of 4 points) over 25 months, indicating considerable speech impairments. However, the participant can still communicate orally without an augmentative communication system, albeit with poor intelligibility to naive listeners.

For a more detailed evaluation of speech deterioration, we analyzed changes in speech acoustics across all experimental sessions. Based on speech spectrograms, we observed that daily medians of the formant frequencies F1 and F2 evolved significantly (p < 0.001) for certain vowels over the two-year study period. Figure 4B visualizes the progression of these formant frequencies for all three vowels, where light-to-dark coloring represents the number of days that have elapsed since electrode implantation. In particular, the phonation of the /a/ vowel showed a trend towards lower frequencies for F1 (slope = −0.084 Hz/day, *P* = 1.488 × 10^− 5^, 95%CI: [−0.121, −0.047], Fig. 4C), while the trend for F2 showed modest increases (slope = 0.055 Hz/day, *P* = 0.00261, 95% CI: [0.020, 0.091], Fig. 4D). We also observed a significant trend in F2 for the /i/ vowel (slope = −0.166 Hz/day, *P* = 1.610 × 10^− 5^, 95% CI: [−0.240, −0.092], Fig. 4D). All these changes in the respective formant frequencies can cause a reduction in the triangular vowel space associated with a decline in speech intelligibility (Fig. 4E, F).[29, 31]

We complemented the evaluation of speech acoustics with regular clinical assessment from a speech-language pathologist. Throughout the study period, biannual consultations reported that the participant presented with a stable mixed spastic-flaccid dysarthria and that diadochokinesis showed no major decline. However, those consultations also revealed variations in the maximum sustained phonation of vowels, consistent with the shifts in formant frequencies observed in the syllable-repetition data.

### ECoG HG responses provided stable offline syllable decoding

**Figure 5:**
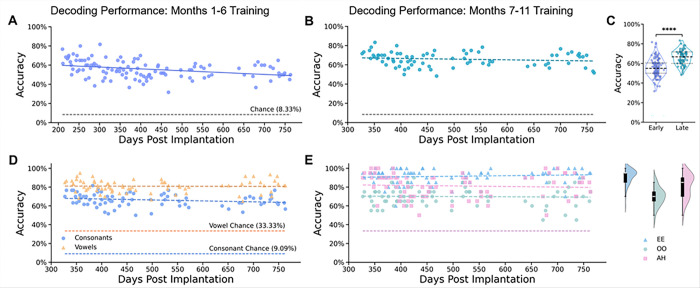
CNN-based syllable decoding performance stability for over a year. (A), (B) Decoding accuracy for models trained on months 1–6 (A) and months 7–11 (B) post-implantation. Each dot represents one day of testing data. Average chance = 8.33%. (C) Violin plots comparing decoding accuracy between early (months 1–6) and late (months 7–11) training periods, with significance (****P< 0.0001, Welch t-test). (D) Vowel-consonant category decoding performance over time, with vowel chance = 33.33% and consonant chance = 9.09%. (E) Vowel phoneme-specific decoding accuracy with corresponding violin plots showing performance distribution across sessions. Vowel chance = 33.33% (light purple dashed line). Solid regression lines indicate statistically significant trends (α < 0.05, two-tailed t-test), while dashed lines indicate non-significance.

Lastly, we evaluated the temporal stability of CNN models trained on HG responses to investigate whether articulation and neural signal quality affected decoding performance. As shown in Fig. 5A, the model trained on the first six months of data achieved relatively stable decoding accuracies (average: 55.67%, 95% CI: [53.99%, 57.34%]) throughout the 554-day test period, which was well above the chance level (8.33%). However, we also observed a significant decline in performance over test sessions in phase 2 (Fig. 5A; slope = −0.0193%/day, *P* = 2.10 × 10^− 4^). In contrast, the model trained on data from months 7–11 (late-trained) showed greater accuracy (mean accuracy: 65.90%, 95% CI: [64.20%, 67.59%]) and had no evident performance decline over time (Fig. 5B; slope = −0.0074%/day, *P* = 0.231). These results suggest that training data after 6 months supported more stable and reliable decoding models. Direct comparisons confirmed an improvement in the accuracy of the late-trained model (Δ = 8.8%, P < 0.001, Fig. 5C).

Confusion matrix analysis (Supplementary Fig. 4A, B) further showed that although the accuracy rates differed, the decoding preferences of the two training models were essentially the same. Misclassifications occurred between syllables with vowels and consonants that shared similar articulatory features (e.g., “THEE” vs. “HEE”, “YAH” vs. “LAH”, “GEE” vs. “JEE”; Supplementary Fig. 5), but there were exceptions (e.g., “TOO” with “GEE”, “ZOO” with “JEE”; Supplementary Fig. 5, 6), which were caused by lingual weakness affecting patient’s ability to articulate these phonemes. Interestingly, “HAH” displayed the most variable waveform across trials yet achieved the highest decoding accuracy in both models, potentially due to its uniquely delayed HG peak (Supplementary Fig. 6). The late-trained model also showed stable results when decoding vowels or consonants alone (vowel slope = −0.0020%/day, *P* = 0.724, consonant slope = −0.0069%/day, *P* = 0.321; Fig. 5D). Phoneme-specific analysis showed that certain vowels (e.g., “EE” and “AH”) were consistently decoded with high accuracy (> 80%), while “OO” showed more variability (70%; Fig. 5E).

To more systematically assess whether the contributions of different electrodes to the speech decoding model remained consistent, we analyzed the saliency distributions of models obtained from training at two stages (Supplementary Methods 5, Supplementary Fig. 7). The results indicated that both models displayed stable spatial preferences (Pearson’s correlation coefficients were all greater than 0.99), with more salient electrodes concentrated primarily in the dorsal lateral region of the array, specifically in the vSMC region associated with the lips.[26] Additionally, the region previously linked to laryngeal movements also showed a moderate contribution.[26] Notably, the key contributing electrode remained essentially stable throughout the follow-up (Supplementary Fig. 7). These results support the relative stability of the lip-larynx motor coding region during disease progression and further explain why the decoding performance remained stable over the long term.

## Discussion

The present study investigated ECoG BCI signal stability in a clinical trial participant with ALS, tracking HG signal characteristics and BCI decoding performance longitudinally over 25 months. Stable neural signal features are a crucial component in developing stable and reliable speech decoding models. Many implantable BCI studies in the literature report their findings on periods that are not representative of long-term performance, including studies based on data acquired in epilepsy monitoring units.[10] Our results provide further insights regarding signal stability in chronic implants and how long-term changes in signal strength and neural responses affect the longevity of BCI speech-decoding models.

By analyzing the regression trend for Power_HG_ (Supplementary Fig. 3, Supplementary Table 1), we noticed that although ActR remained relatively stable over the 19-month period (Fig. 3A), this stability was accompanied by comparable declines in HG band power during both baseline and active periods (Fig. 3B, Supplementary Fig. 3). The decline in Power_HG_ could have been due to alterations at the electrode-tissue interface. Previous studies suggest that ALS progression causes progressive brain atrophy, potentially increasing the distance between the cortical surface and electrodes and altering the electrode-tissue interface.[19] We also observed that the z-scored HG peak (the normalized maximum HG power during vocalization) gradually increased over time; however, this may not mean that more neurons were recruited or that neuronal activity became more intense during vocalization. Since the peak of the Power_HG_ was also gradually decreasing, the more likely explanation for the rising z-scored HG peak is that the rate of decline in the absolute peak was slower than the rate of decrease in the baseline (Supplementary Fig. 3, Supplementary Table 1). We examined the full width at half maximum of the z-scored HG waveforms, which showed a decreasing trend, indicating that the response waveforms become narrower (Supplementary Fig. 8). This may be due to the patient becoming more familiar with the task or to a neural compensatory mechanism that developed to keep vocal output as consistent as possible despite disease progression affecting articulation ability.[38, 39] For example, the remaining healthy neurons in the cortex may have been driven more strongly or fire more synchronously during speech.[39]

Similar to previous work with an ALS patient, the HG activity first improved within the first few months before gradually decreasing.[23, 40] While the subsequent downward trend was not entirely consistent with previous findings in non-ALS participants, where HG power showed strong stability,[27, 41] this decrease was similar to the progressive attenuation of ECoG band power (65–95Hz) in the primary motor cortex observed during baseline periods in another study for an individual with ALS.[18, 19] Notably, the decrease in SNR was pronounced in the resting state, with little long-term change in HF noise activity, suggesting that the diminished neural activity was not entirely attributable to noise fluctuations.

We found that while the absolute amplitude of HG responses (Power_HG_) diminished over time, temporal structures of the z-scored HG waveforms remained consistently stable across 12 different syllables after the first 6 months (averaged time-envelope root-mean-square error (RMSE) < 0.6 SD, Supplementary Fig. 9, Supplementary Methods 6). The preservation of this relative neural pattern is crucial, as it indicates that the underlying neural features are largely unaffected by overall signal decay.[23] Stability of decoding accuracy has important implications for the clinical feasibility of neural prostheses, suggesting that ECoG-based systems can withstand a certain level of neurodegenerative processes and may not require the frequent and cumbersome recalibration typically considered necessary. This aligns with numerous previously reported results showing that long-term ECoG decoding is robust, even under degrading conditions. [5, 11, 18, 42]

Through acoustic analysis of formant frequencies, we observed a mild reduction in subjects’ speech intelligibility, but it was not reflected in the clinically used ALSFRS-R scores. The reduction in VSA led to decreased vowel differentiation, reflecting lingual weakness, while F1 and F2 changes were directly related to tongue height and anterior-posterior movement, all of which serve as markers of ALS progression.[43, 44] The progressive functional decline of the articulatory muscles (lingual weakness) may pose a long-term challenge for decoding. As muscle control degrades, it may affect a participant’s ability and way to articulate these syllables. The confusion matrix of our decoder suggests confusion between acoustically similar syllables, possibly due to similar neural responses (Supplementary Fig. 6, 9). Disease progression may deepen this similarity,[7, 45] which provides new insights into the future direction of speech BCIs for ALS patients. For example, besides making algorithmic improvements, one could consider selecting words or commands based on their ability to produce more distinct neural representations, rather than relying solely on their acoustic differences. This approach would directly target the decoding confusion that occurs when neural signals for different phonemes in overt speech start to converge as the disease progresses.[11] Furthermore, saliency analysis revealed that neural signals recorded from potential lip representations contributed more to decoding than those from potential tongue or jaw representations.[11, 26] By examining three matrices used to measure signal stability, we found that lip area exhibits higher stability in this participant, particularly evident in z-scored HG peaks. This may reflect differences in atrophy rates across vocal musculature.

An important limitation of our study is that we cannot rule out an effect from changes in impedance; Hence, the signal attenuation observed in ALS may result from a combination of impedance changes and the progressive loss of motor neurons.[46, 47] Another important limitation of this study is that there was only one participant with slowly progressive disease and moderate dysarthria, and all conclusions still need to be validated with participants who present with a wider range of disease trajectories and speech/oral motor impairment. Given the heterogeneity of ALS and the nonlinear course of the disease, replication of the study in a larger cohort with varying disease durations may be needed to generalize our findings.[48] As our participant had an eight-year history of the disease at the time of implantation, the ability to effectively maintain HG dynamics and stable decoding in patients whose disease is more rapidly progressing remains a question worth exploring. Beyond the single-case design, several other limitations should be considered. First, our analysis relied on offline decoding, and integrating real-time decoding is essential to verify the clinical usefulness of the observed trends in a functional BCI environment. Second, the study employed a highly controlled syllable repetition task. While this approach effectively measures changes in specific neural signals, it is far from natural, spontaneous, and everyday communication. Real conversations involve complex grammar, varying speech rates, nonverbal intentions, and a much larger vocabulary. Therefore, the decoding stability seen in a restricted task might not directly translate to free conversation contexts. On the other hand, our study did not use a language model, which has been required for recent demonstrations of sentence decoding. Not using a language model allowed us to focus on longitudinal trends in decoding performance and how these relate to longitudinal trends in signal quality.

Looking ahead, this study highlights the potential of ECoG-based speech neural prostheses as a long-term communication solution for ALS patients. Its broader implications lie in showing that functional decoding can be preserved even with longitudinal reductions in neural signal strength. The next essential step is to incorporate these insights into a long-term, online, closed-loop system so participants can use it for real-time communication in their daily lives. Ultimately, creating decoding algorithms that can learn and adapt to the slow signal changes observed here could further improve the longevity and reliability of BCI systems, bringing us closer to providing truly sustainable communication methods for those who have lost their ability to speak.

## Supplementary Material

Supplementary Files

This is a list of supplementary files associated with this preprint. Click to download.

• SupplementaryMaterial1.pdf

## Data Availability

Anonymized data not published in this article will be made available upon request from any qualified investigator.
